# Longitudinal Cohort Study Investigating Fall Risk Across Diverse Muscle Health Statuses Among Older People in the Community

**DOI:** 10.1002/jcsm.13788

**Published:** 2025-03-31

**Authors:** Yuan‐Ping Chao, Wen‐Hui Fang, Tao‐Chun Peng, Li‐Wei Wu, Hui‐Fang Yang, Tung‐Wei Kao

**Affiliations:** ^1^ Division of Family Medicine, Department of Family and Community Medicine Tri‐Service General Hospital; and School of Medicine, National Defense Medical Center Taipei Taiwan; ^2^ Division of Geriatric Medicine, Department of Family and Community Medicine Tri‐Service General Hospital, National Defense Medical Center Taipei Taiwan

**Keywords:** dynapenia, fall, possible sarcopenia

## Abstract

**Background:**

Falls constitute a significant public health concern among older adults, particularly those with diminished muscle health integrity. The relative contributions of reduced muscle mass versus impaired muscle function to fall risk remain debated. Discrepant conclusions in previous studies exist due to divergent muscle health categorizations and parameter measurements. This study investigated longitudinal risk of falls across a spectrum of muscle health statuses among older people in the community.

**Methods:**

Community‐dwelling participants aged 65 years and older, undergoing annual health assessments, were enrolled between 2015 and 2023. Measurements included handgrip strength, walking speed and appendicular skeletal muscle mass. Dynapenia was defined as impaired muscle function with preserved muscle mass, presarcopenia was characterized as reduced muscle mass with maintained muscle function, and sarcopenia was identified as concurrent losses in both muscle mass and muscle function. Older people with normal muscle mass and muscle function were defined as having a robust muscle health status. Participants without a history of falls at baseline were monitored continuously and were censored if a fall incident was recorded during later yearly assessment. Kaplan–Meier and Cox regression analyses were used to compare fall risk across different muscle health statuses.

**Results:**

The final analysis included a total of 863 participants with a mean age of 71.93 ± 6.62 years, and 57.58% were female. Compared with the other groups, the dynapenic group exhibited a lower physical activity, greater body mass index and slower gait speed. The participants with dynapenia experienced the highest fall incidence (27.15%). The hazard ratios (HRs) for fall were 2.65 (95% confidence interval [CI] = 1.72–4.08, *p* < 0.001) for dynapenia, 1.54 (95% CI = 0.92–2.57, *p* = 0.095) for presarcopenia and 1.87 (95% CI = 1.04–3.33, *p* = 0.034) for sarcopenia. After adjustment for multiple covariates, the fall risk remained significantly greater in the dynapenic group (HR = 2.10, 95% CI = 1.28–3.43, *p* = 0.003) than in the sarcopenic group (HR = 1.31, 95% CI = 0.69–2.46, *p* = 0.402). Female dynapenic participants with coronary artery disease, arthritis and sedative agent use had a high fall risk, especially those with two or more risk factors (HR = 2.82, 95%CI = 1.37–5.82, *p* = 0.005).

**Conclusions:**

Older adults with dynapenia exhibited a greater fall risk than did those with sarcopenia. Dynapenic older people with two or more risk factors, such as female sex, coronary artery disease, arthritis and sedative agent use, may have an increased longitudinal fall risk. Promoting muscle function should be prioritized as a preventive strategy to mitigate adverse clinical outcomes.

## 1 Introduction

The term ‘sarcopenia’ was originally introduced to describe the age‐associated decline in skeletal muscle mass, with an initial focus solely on the reduction in muscle mass, without consideration of muscle strength or functional limitations [1]. The first clinical operational definition of sarcopenia was established in 2010, highlighting the importance of muscle mass loss in conjunction with functional decline [2]. Nevertheless, prior to the exploration of this consensus, the concept of dynapenia, defined as a decline in muscle strength and/or power, began to gain recognition in 2008 [3]. Clark and colleagues argued that muscle loss is not the predominant factor in age‐related strength decline. Instead, they suggested that the emphasis should be redirected towards understanding the decline in muscle strength itself [4]. Muscle health fundamentally comprises two key components: muscle quantity, which pertains to muscle mass, and muscle quality, which encompasses aspects such as muscle strength and physical performance. The impact of muscle health on clinical outcomes varies, indicating that both muscle quantity and quality should be assessed concurrently.

Despite the evolving definitions of sarcopenia and dynapenia, both muscle mass loss and functional decline have been linked to an increased risk of falls. Veronese and colleagues, drawing on data from the Study on Global Ageing and Adult Health, highlighted that sarcopenia is significantly associated with an increased risk of fall‐related injuries. Potential mechanisms may involve impaired balance, reduced gait speed and hormonal imbalances associated with sarcopenia [5]. Conversely, Smith and colleagues, utilizing data from the Irish Longitudinal Study on Ageing survey, reported that dynapenia is linked to an 8% increased risk of falls. Additionally, individuals with dynapenic abdominal obesity (DAO) exhibit a 47% increased risk of falls [6]. On the other hand, some studies have proposed that fall and fracture risk predicted on the basis of reduced muscle mass may exhibit sex differences [7, 8]. Nonetheless, another study showed that declines in handgrip strength and lower limb function are more dependable indicators of the risk of falls and fractures [9]. Consequently, Scott and colleagues proposed that markers of dynapenia might offer a more accurate assessment of fall and fracture risk in older adults than markers of sarcopenia. As a result, divergent findings across various studies have led to controversy. Nevertheless, no studies have yet utilized expert consensus standards to define various muscle health statuses and assess their associations with long‐term fall risk.

Given that muscle health encompasses both muscle mass and function, older individuals can present with varying muscle health statuses—such as a robust status, dynapenia, presarcopenia and sarcopenia—depending on the combinations of these parameters. We aimed to resolve this clinical ambiguity and hypothesized that a decline in muscle function has a more substantial effect on fall risk than does muscle mass loss alone or the loss of both muscle mass and muscle function with increasing age. This longitudinal study included community‐dwelling individuals aged 65 years and older in northern Taiwan. By employing the muscle health parameter cut‐off values defined by the Asia Working Group on Sarcopenia (AWGS) in 2019, this study investigated future fall risk across diverse muscle health statuses. The contributions of various fall risk factors associated with these muscle health statuses were estimated in this prospective study as well.

## 2 Methods

### 2.1 Participants

From March 2015 to July 2023, we enrolled community‐dwelling adults aged 65 years and older who underwent annual health examinations. The exclusion criteria included congestive heart failure, chronic kidney disease necessitating regular haemodialysis, cognitive impairment, malignancies requiring ongoing medical treatment or a history of chest or bone pain associated with physical exertion. An investigator employed a structured questionnaire to gather data on the demographic profile, general health status and daily physical activity levels of all participants. All participants were required to provide informed consent prior to their involvement in the study, which was approved by the Institutional Review Board of Tri‐Service General Hospital (TSGHIRB Number: 2‐103‐05‐024). The participants' demographic and lifestyle information, including age, sex, cigarette smoking status, alcohol consumption status and medical history, was initially obtained through a self‐administered questionnaire and subsequently verified against health insurance cards. Detailed histories of specific medical conditions such as hypertension, diabetes mellitus, stroke, coronary artery disease and arthritis were recorded on the basis of participants' self‐reports of previous diagnoses by their physicians. Physical activity was assessed via the Chinese version of the International Physical Activity Questionnaire (IPAQ) [10]. For biochemical analyses, blood samples were collected after an 8‐h fasting period to measure total cholesterol and serum albumin levels. The International Classification of Diseases (ICD‐10) codes of considerable comorbidities or health conditions in this study were listed in Table S1.

### 2.2 Exploration of Participants' Body Compositions

The participants' height was measured with a stadiometer, and their weight was assessed via a digital scale. Body mass index (BMI) was computed as weight in kilograms divided by the square of height in meters. Waist circumference (WC) was determined at the midpoint between the lower edge of the 12th rib and the top of the iliac crest, with participants standing and their feet spaced 25 to 30 cm apart.

Appendicular skeletal muscle (ASM) comprises the lean muscle mass of the arms and legs, quantified via an eight‐electrode bioelectrical impedance analyser (BIA) (InBody 720, Biospace, Seoul, South Korea). The skeletal muscle mass index (SMI), denoted as the ASM adjusted for height squared (ASM/ht^2^), was calculated to assess skeletal muscle mass. Additionally, the BIA was employed to assess the participants' body fat mass and body fat percentage. The fat‐to‐muscle ratio (FMR) was calculated as the quotient of body fat mass divided by body muscle mass. According to the 2019 consensus established by the AWGS, the defined cut‐off value for a low SMI was 7.0 kg/m^2^ in men and 5.7 kg/m^2^ in women [11].

### 2.3 Muscle Strength and Walking Speed Assessment

The handgrip strength of the participants' dominant hands was assessed three times via an analogue isometric dynamometer (Exacta™ Hydraulic Hand Dynamometer; North Coast Medical Inc., Gilroy, CA), with the highest value recorded. For the walking test, the participants were instructed to walk 10 m at their usual pace, and the length of time it took for them to pass 6 m in between was measured; gait speed was then calculated as the ratio of walking distance (meters) to walking time (seconds). According to the 2019 AWGS criteria, low handgrip strength was indicated as a value below 28 kg for older men and less than 18 kg for older women. A gait speed slower than 1.0 m/s was considered low for both sexes.

### 2.4 Determination of Muscle Health Status

Muscle health encompasses three parameters including muscle mass, muscle strength and physical performance. Older adults exhibiting normal muscle health as measured by the SMI, handgrip strength and gait speed were classified as having a robust status. Those with low handgrip strength and/or a low gait speed but a normal SMI were classified as having dynapenia. Participants with normal handgrip strength and a normal gait speed but a low SMI were categorized as having presarcopenia. Sarcopenia was defined as a low SMI in conjunction with low handgrip strength, or a low gait speed, or both. We adhered to the 2019 consensus of the AWGS [11] to establish all cut‐off points for a low SMI, low handgrip strength and a slow gait speed.

### 2.5 Data Collection and Statistical Analysis

Between 2015 and 2023, baseline assessments and annual observations were conducted by well‐trained research assistants. The fall was defined as ‘an unexpected event in which the participant comes to rest on the ground, floor, or lower level’, which was established by the international Fall Outcomes Consensus Group, the Prevention of Falls Network Europe [12]. Throughout the follow‐up period, participants were censored if they had experienced a fall event within the preceding year. Continuous variables are presented as the means ± standard deviations, whereas categorical data are expressed as numbers with percentages. An ANOVA test was conducted to examine differences in covariates across various muscle health statuses. Previous studies [6, 13] have reported effect sizes for fall risk ranging from 1.47 to 1.7, with the proportion of falls estimated between 0.25 and 0.33. Informed by these findings, the sample size for this study was calculated using a significance level (α) of 0.05 and a statistical power (1 − β) of 0.8, following the methodology described by Schoenfeld [14]. To address the inflation of Type I error inherent in multiple testing (six pairwise comparisons across four groups of muscle health status), a Bonferroni‐adjusted significance threshold of *p* < 0.008 was applied. The associations between muscle health status and the cumulative hazard of fall events were assessed via Kaplan–Meier analysis. Cox regression analysis was employed to evaluate the risk of fall events among individuals with dynapenia, presarcopenia and sarcopenia. An extended model approach incorporating stepwise variable adjustment via Cox proportional hazards regression was implemented. Various covariate adjustments were applied as follows for fall risk estimation: Model 1 included age, sex, smoking status and alcohol consumption status; Model 2 included all variables from Model 1 plus metabolic syndrome, stroke, coronary artery disease, arthritis, osteoporosis and physical activity level; Model 3 included all variables from Model 2 plus depression and the use of antipsychotic and sedative agents. In further subgroup analysis, the hazard of risk factors contributing to fall events among participants with dynapenia was assessed via Cox regression analysis. The identification of dynapenic individuals at high‐risk for fall events was determined via receiver operating characteristic (ROC) curve analysis. All analyses were conducted via the Statistical Package for the Social Sciences (SPSS), version 18.0 (SPSS, Inc., Chicago, IL).

## 3 Results

### 3.1 Characteristics of Participants With Different Muscle Health Statuses

**FIGURE 1:**
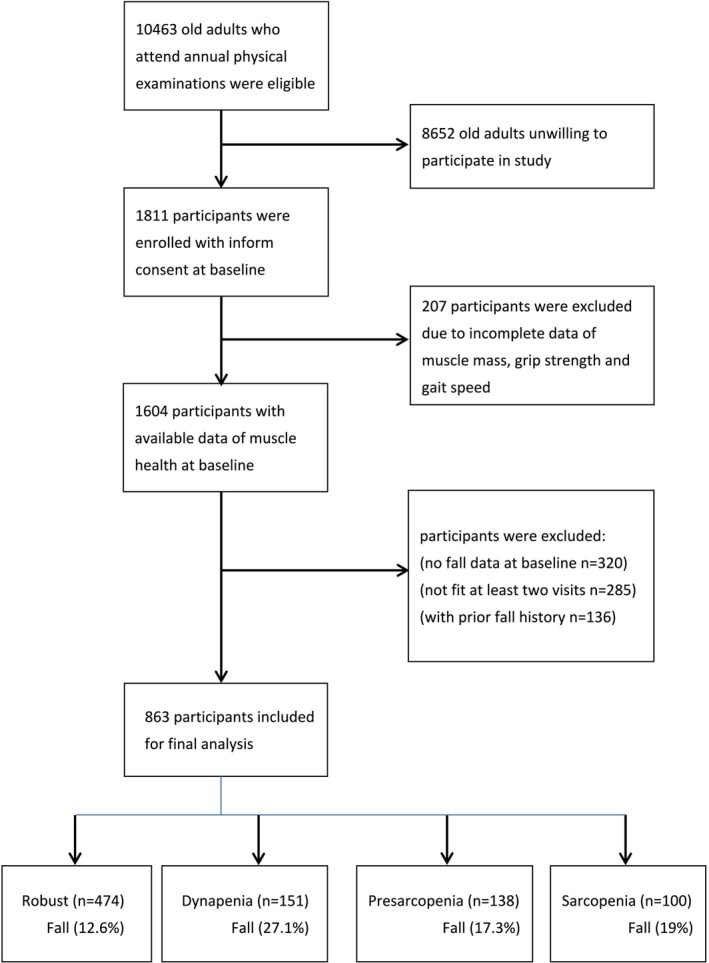
Flow chart of participants' enrollment.

Between 2015 and 2023, a total of 10 463 elderly patients underwent annual routine health examinations at our medical centre and were deemed eligible for the study. Upon receiving detailed information regarding the purpose of this study, 8652 individuals declined to participate, resulting in the inclusion of 1811 elderly patients. Additionally, 948 participants were excluded because of incomplete muscle health parameters, missing baseline data on falls, failure to meet the criterion of a minimum of two follow‐up visits or a pre‐existing history of falls. Consequently, the final cohort for the longitudinal fall risk analysis, stratified by muscle health status, comprised 863 participants (Figure 1).

Among the total participants, the mean age was 71.93 ± 6.62 years, and 57.58% of the participants were females. The median follow‐up duration was 23.85 months, with an interquartile range of 13.24 to 48.59 months. Compared with the other groups, the dynapenic group exhibited higher blood pressure, lower physical activity levels and a slower gait speed. Dynapenic older adults had higher values of obesity indicators, including BMI, WC, body fat mass and body fat percentage. Dynapenic older adults were also more likely to have hypertension, diabetes mellitus and metabolic syndrome than older adults with other muscle health statuses (Table 1).

**TABLE 1:** Characteristics of the participants categorized by different muscle health statuses.

Covariates	Robust (*n* = 474)	Dynapenia (*n* = 151)	Presarcopenia (*n* = 138)	Sarcopenia (*n* = 100)	*p*
Continuous variables^a^					
Age (years)	70.31 ± 5.08	73.93 ± 7.96	71.60 ± 6.13	77.10 ± 8.00	< 0.001
BMI (kg/m^2^)	25.06 ± 3.04	25.58 ± 3.35	21.49 ± 2.28	21.91 ± 2.80	< 0.001
WC (cm)	82.37 ± 8.79	83.25 ± 9.92	73.67 ± 7.48	76.19 ± 7.79	< 0.001
SBP (mmHg)	133.64 ± 15.36	133.67 ± 18.23	130.33 ± 18.56	132.76 ± 18.51	0.223
Grip strength (kg)	30.45 ± 8.64	19.86 ± 7.48	25.56 ± 7.09	17.60 ± 6.16	< 0.001
Gait speed (m/s)	1.60 ± 0.35	1.19 ± 0.45	1.65 ± 0.34	1.25 ± 0.41	< 0.001
Physical activity (kcal/week)	10127.45 ± 3330.01	9257.19 ± 3508.97	9711.90 ± 2716.38	9391.23 ± 2672.02	0.012
SMI (kg/m^2^)	7.06 ± 0.91	6.91 ± 0.97	5.70 ± 0.70	5.58 ± 0.73	< 0.001
Fat mass (kg)	19.77 ± 5.94	21.14 ± 6.71	15.76 ± 4.13	16.87 ± 5.54	< 0.001
Body fat percentage (%)	30.37 ± 7.25	32.52 ± 8.06	30.21 ± 6.47	31.71 ± 7.86	0.011
Fat‐to‐muscle ratio	0.84 ± 0.30	0.95 ± 0.37	0.84 ± 0.27	0.96 ± 0.33	0.001
Haemoglobin (g/dL)	13.83 ± 1.15	13.46 ± 1.41	13.42 ± 1.24	12.99 ± 1.52	< 0.001
LDL (mg/dL)	109.80 ± 29.47	103.36 ± 30.31	114.00 ± 32.23	105.13 ± 28.09	0.018
Albumin (mg/dL)	4.32 ± 0.22	4.27 ± 0.25	4.34 ± 0.23	4.23 ± 0.26	0.001
Categorical variables^b^					
Male	232 (48.9)	60 (39.7)	43 (31.1)	31 (31)	< 0.001
Smoker	116 (24.4)	20 (13.2)	19 (13.7)	15 (15)	0.002
AC ≥ 4 times/month	50 (10.5)	4 (2.6)	7 (5.1)	2 (2)	0.001
Metabolic syndrome	104 (21.9)	42 (27.8)	8 (5.7)	10 (10)	< 0.001
Hypertension	159 (33.5)	72 (47.6)	32 (23.1)	41 (41)	< 0.001
Diabetes	57 (12.0)	34 (22.5)	11 (7.9)	14 (14)	0.002
Stroke	7 (1.4)	7 (4.6)	1 (0.7)	5 (5)	0.020
CAD	23 (4.8)	10 (6.6)	7 (5.1)	6 (6)	0.837
Arthritis	79 (16.6)	29 (19.2)	19 (13.7)	23 (23)	0.228
Osteoporosis	33 (6.9)	20 (13.2)	21 (15.2)	16 (16)	0.003
Depression	11 (2.3)	11 (7.2)	13 (9.4)	2 (2)	< 0.001
Antipsychotics	3 (0.6)	2 (1.3)	0 (0)	2 (2)	0.312
Sedative agents	74 (15.6)	30 (19.8)	33 (23.9)	16 (16)	0.124

^a^ Values in the continuous variables were expressed as mean ± standard deviation unless otherwise specified.

^b^ Values in the categorical variables were expressed as number (percent).

Abbreviations: AC, alcohol consumption; BMI, body mass index; CAD, coronary artery disease; HDL, high‐density lipoprotein; LDL, low‐density lipoprotein; SBP, systolic blood pressure; SMI, skeletal muscle mass index; TG, triglyceride; WC, waist circumference.

### 3.2 Estimation of Fall Risk Among Older Adults With Different Muscle Health Statuses

**FIGURE 2:**
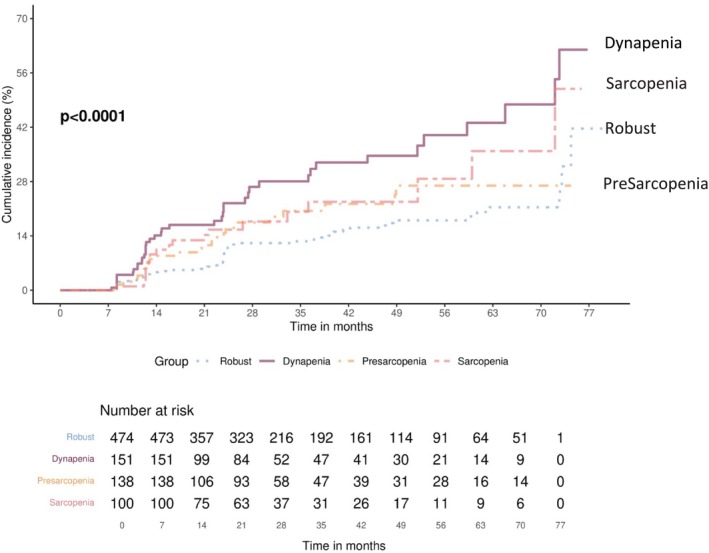
Accumulative risk of fall event among participants with different muscle health statuses.

Among the included older adults, those with dynapenia had the highest incidence of falls (27.1%), whereas those with a robust status had the lowest incidence of falls (12.6%) (Table 2). The cumulative risk of fall events among dynapenic participants was greater than that among sarcopenic individuals and individuals with other muscle health statuses according to Kaplan–Meier analysis (Figure 2). The hazard ratios (HRs) for fall events were 2.65 (95% confidence interval [CI] = 1.72–4.08, *p* < 0.001) for the dynapenic group, 1.54 (95% CI = 0.92–2.57, *p* = 0.095) for the presarcopenic group and 1.87 (95% CI = 1.04–3.33, *p* = 0.034) for the sarcopenic group. After adjustment for multiple covariates, the fall risk remained significantly greater in the dynapenic group (HR = 2.10, 95% CI = 1.28–3.43*, p* = 0.003) than in the sarcopenic group (HR = 1.30, 95% CI = 0.69–2.46, *p* = 0.402).

**TABLE 2:** Hazard ratio of fall event among participants with different muscle health statuses.

	Robust (*n* = 474)	Dynapenia (*n* = 151)		Presarcopenia (*n* = 138)		Sarcopenia (*n* = 100)	*p*
Fall, *n* (%)	60 (12.6)	41 (27.1)		24 (17.3)		19 (19)	< 0.001
		HR (95% CI)	*p* ^b^	HR (95% CI)	*p* ^b^	HR (95% CI)	*p* ^b^
Unadjusted	Ref.	2.65 (1.72–4.08)	< 0.001	1.54 (0.92–2.57)	0.095	1.87 (1.04–3.33)	0.034
Adjusted models^a^							
Model 1	Ref.	2.10 (1.31–3.37)	0.002	1.27 (0.75–2.14)	0.359	1.29 (0.69–2.40)	0.412
Model 2	Ref.	2.08 (1.27–3.40)	0.003	1.29 (0.75–2.20)	0.349	1.29 (0.69–2.42)	0.421
Model 3	Ref.	2.10 (1.28–3.43)	0.003	1.27 (0.73–2.20)	0.387	1.30 (0.69–2.46)	0.402

Abbreviations: CI, confidence interval; HR, hazard ratio; Ref, reference.

^a^ Adjusted covariates. Model 1 = age, sex and health behaviours (smoking and alcohol consumption), Model 2 = Model 1+ metabolic syndrome, stroke, coronary artery disease, arthritis, osteoporosis and physical activities. Model 3 = Model 2+ depression, use of anti‐psychotic agents and sedative agents.

^b^ The Bonferroni corrected values of *p* < 0.008 were considered statistically significant.

### 3.3 Factors Contributing to Fall Events in Dynapenic Older People

The study estimated the HRs of various covariates for fall events among participants with dynapenia. The results revealed that male participants with dynapenia had a lower HR for fall events (HR = 0.50, 95% CI = 0.25–0.99, *p* = 0.048). Older people with coronary artery disease presented a greater risk of experiencing fall event (HR = 2.46, 95%CI = 1.02–5.87, *p* = 0.043). Individuals with arthritis tended to have a greater fall risk (HR = 2.04, 95% CI = 1.04–3.98, *p* = 0.036). Older adults who used sedative agents were more likely to experience a fall event (HR = 2.95, 95%CI = 1.51–5.76, *p* = 0.001) (Table 3).

**TABLE 3:** Hazard of covariates to fall event among participants with dynapenia.

Covariates	Hazard ratio (95% confidence interval)	*p*
Age	1.02 (0.98–1.06)	0.224
Male	0.50 (0.25–0.99)	0.048
smoking	0.77 (0.27–2.16)	0.619
AC ≥ 4 times/month	0.79 (0.11–5.81)	0.820
Body mass index	0.97 (0.88–1.07)	0.596
Waist circumference	0.99 (0.97–1.02)	0.839
Hypertension	1.11 (0.59–2.05)	0.741
Diabetes mellitus	1.73 (0.86–3.50)	0.123
Stroke	1.91 (0.58–6.22)	0.282
Coronary artery disease	2.46 (1.02–5.87)	0.043
Arthritis	2.04 (1.04–3.98)	0.036
Osteoporosis	1.20 (0.49–2.88)	0.684
Physical activity	1.00 (1.00–1.00)	0.757
Depression	1.76 (0.54–5.77)	0.346
Anti‐psychotics	1.95 (0.26–14.31)	0.512
Sedative agents	2.95 (1.51–5.76)	0.001
Body fat percentage	1.04 (0.99–1.08)	0.074
Fat‐to‐muscle ratio	1.92 (0.75–4.94)	0.172
Haemoglobin	0.76 (0.57–1.01)	0.057
LDL	0.99 (0.98–1.00)	0.250
Albumin	0.55 (0.16–1.80)	0.325

Abbreviations: AC, alcohol consumption; LDL, low‐density lipoprotein.

### 3.4 High and Low Risk of Fall Events in Dynapenic Individuals

**FIGURE 3:**
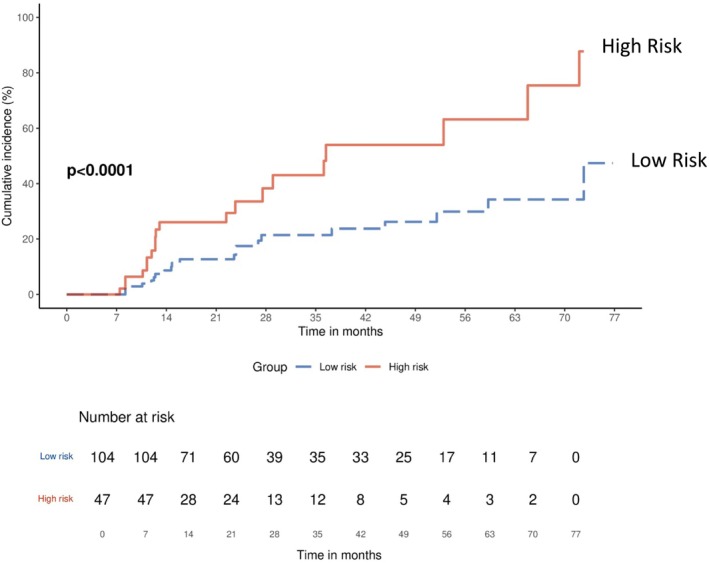
Accumulative risk of fall event between dynapenic participants with high risk factors and low risk factors.

Among participants with dynapenia, four risk factors contributed to the risk of future fall events. We subsequently categorized participants into high‐risk and low‐risk groups based on the presence of two or more risk factors. The cumulative risk of fall events in high‐risk group of dynapenic participants was greater than those in low‐risk group according to Kaplan–Meier analysis (Figure 3). Cox regression analysis revealed that high‐risk group of dynapenic participants had a greater risk of fall event than those of low‐risk group (HR = 2.48, 95%CI = 1.29–4.74, *p* = 0.006). The greater risk for fall events remained after adjusting for multiple confounding covariates (HR = 2.82, 95%CI = 1.37–5.82, *p* = 0.005) (Table 4).

**TABLE 4:** Hazard ratio of fall event among dynapenic participants with high risk factors.

	Hazard ratio (95% confidence interval)	*p*
Unadjusted	2.48 (1.29–4.74)	0.006
Adjusted models^a^		
Model 1	2.59 (1.32–5.08)	0.005
Model 2	2.72 (1.33–5.59)	0.006
Model 3	2.82 (1.37–5.82)	0.005

^a^ Adjusted covariates. Model 1 = age, smoking and alcohol consumption. Model 2 = Model 1+ metabolic syndrome, stroke, osteoporosis and physical activities. Model 3 = Model 2+ depression and use of anti‐psychotic agents.

## 4 Discussion

This study demonstrated that individuals with dynapenia had a greater cumulative fall risk than did those with sarcopenia throughout the 8‐year period. Falls in older adults are attributable to the interplay of age‐related physical, sensory and cognitive changes, compounded by environmental factors that are not conducive to their safety [15]. Although the causality of falls is multifactorial, in meta‐analyses of fall risk studies, the tools employed to assess falls predominantly focus on functional evaluations and generally do not address the reduction in muscle mass [16]. This finding can be interpreted from multiple perspectives. First, a reduction in muscle strength does not necessarily correspond to a loss of muscle mass [17]. Even when both muscle mass and strength are reduced, their rates of decline are not synchronized, with muscle strength typically waning in a more swift manner than muscle mass does [18, 19]. Visser et al. reported that reduced muscle strength is a more significant determinant of fall risk than reduced muscle mass is. [20]. Newman et al. demonstrated a strong correlation between muscle strength and mortality in elderly individuals, which was somewhat attenuated when muscle mass was accounted for [21]. In our study, in the group of individuals with presarcopenia, which was characterized solely by muscle mass loss, the adjusted risk of falls did not significantly increase. These findings indicate that low muscle mass has a minimal influence on the risk of falls. Nevertheless, in the group of individuals with dynapenia, which was characterized by a decline in muscle function alone, the risk of falls was even greater than that in the group of sarcopenic individuals, who exhibited both muscle mass loss and muscle function decline. In addition, skeletal muscle mass has less predictive value than muscle strength in the prediction of disability progression and adverse health outcomes [21]. Furthermore, Clark et al. suggested that dynapenia should be recognized as a distinct phenomenon that is potentially more functionally significant and relevant than sarcopenia is [22, 23]. Our findings indicate that, when accounting for muscle mass loss, both presarcopenia and sarcopenia are less strongly associated with fall risk than dynapenia is, thereby underscoring the pivotal role of dynapenia in functional assessment.

In this study, we also observed that the dynapenic group presented higher values of obesity indicators, including BMI, WC, fat mass and body fat percentage. Some studies suggest that the interplay of pro‐inflammatory cytokines, oxidative stress and insulin resistance creates a bidirectional relationship between muscle weakness and abdominal obesity, which further impairs motor neuron function [24]. Recent large cohort studies have focused on DAO, primarily using WC to define obesity and assess its clinical impact. These studies hypothesized that DAO is associated with poorer functional activity and grouped participants into categories of robust status, dynapenia/nonabdominal obesity (D/NAO), nondynapenia/abdominal obesity (ND/AO) and DAO for functional evaluation. Data from the Irish Longitudinal Study on Ageing (TILDA) revealed that DAO significantly increased the 2‐year fall risk [6], whereas the China Health and Retirement Longitudinal Study (CHARLS) revealed that DAO was associated with a significantly greater risk of functional impairments, with the odds ratios (ORs) for functional impairments in individuals with D/NAO exceeding those for functional impairments in individuals with ND/AO [25]. The English Longitudinal Study of Ageing (ELSA) revealed that over a 14‐year follow‐up, the HRs for falls and repeated falls in the DAO group were 1.195 and 1.276, respectively, with the HRs for both being greater in the D/NAO group than in the ND/AO group [26]. Scott et al. compared 5‐year fall risks between sarcopenic and dynapenic obesity in older adults, defining sarcopenia as solely low muscle mass, similar to our presarcopenia category. Their results revealed that only dynapenic obesity was linked to an increased fall risk [27]. These studies underscore the synergistic effect of dynapenia combined with obesity on adverse clinical outcomes. Furthermore, dynapenia alone, even in the absence of obesity, has a significant effect on adverse outcomes. In our study, we addressed muscle status by categorizing participants into robust, dynapenia, presarcopenia and sarcopenia groups, while tracking various obesity parameters and fall risks over the long term. This approach not only corroborated and extended previous findings but also elucidated the roles of muscle mass and function in these outcomes.

Regarding potential risk factors for falls among individuals with dynapenia, using sedative medications, being an older female adult and having a history of coronary artery disease or arthritis are four risk factors linked to a greater longitudinal fall risk. Participants with two or more risk factors had a 2.82‐fold increased risk of falls throughout the long‐term observation period. Multiple comorbidities appear to be associated with adverse clinical outcomes across different muscle and fat compositions. A cohort study in the United Kingdom revealed that 55% of older participants developed multiple diseases over 10 years, with individuals with DAO and abdominal obesity having ORs of 1.671 (*p* = 0.002) and 1.505 (*p* < 0.001), respectively [28]. Systematic reviews have also highlighted the associations between various forms of arthritis and falls [29]. A sex‐specific pattern was observed in the US Osteoarthritis Initiative study: The OR for functional limitations in men with low muscle strength was 0.98 (95% CI = 0.66–1.46), whereas that in women was 1.60 (95% CI = 1.15–2.22) [30]. These findings align with our study results, underscoring the need for further exploration of the complex associations involved.

Coronary artery disease significantly impacts musculoskeletal health through shared regulatory mechanisms, and handgrip strength has emerged as a key predictor of cardiovascular risk [31, 32]. A longitudinal study conducted in Perth in Australia revealed that women with coronary artery disease had weaker handgrip strength and a slower gait speed, which worsened muscle function and increased fall risk by 25% for each standard deviation decrease in muscle strength [33]. Conversely, high‐intensity interval training (HIIT) has been shown to enhance both muscle strength and cardiovascular health in elderly individuals [34]. Our study demonstrated that coronary artery disease increases the risk of falls in individuals with dynapenia, corroborating existing research.

Sedative medications are a well‐established risk factor for falls in elderly individuals [35], with a greater number of medications increasing this risk due to impaired metabolism [36]. In addition to sleep aids, medications such as antiparkinsonism drugs, opioids, antiepileptics, antipsychotics, antidepressants and muscle relaxants, all increase fall risk because of their sedative effects [37]. Conversely, discontinuing these medications may improve handgrip strength and balance [38]. Our study confirms this risk for falls in dynapenic individuals, which is consistent with previous research.

Muscle strength and physical performance differ between older men and women [39]. Sun et al. reported that the association between DAO and frailty progression varies by sex, with men reported to progress faster in the CHARLS, whereas women were reported to progress faster in the ELSA [40]. Using CHARLS data, Qian et al. identified sex‐specific functional impairments within the dynapenic population [25]. In an ELSA‐based cohort study, Dowling et al. reported that over 2 years, DAO significantly increased fall risk in men, whereas in women, fall risk was driven primarily by dynapenia [13]. Ethnicity, age at sampling, follow‐up duration and sample size may influence sex differences. In our study, men had a 50% lower fall risk than women did, which is consistent with other cohort studies.

To our knowledge, this is the first study to elucidate potential risk factors for falls and integrate them to distinguish thresholds of greater fall risk among community‐dwelling older adults. These findings enhance the ability to identify high‐risk populations, allowing for more targeted interventions in clinical practice. The observational design and reliance on self‐reported data may introduce recall bias, necessitating consideration of this limitation in the study. Moreover, despite thorough statistical adjustments, the influence of potential confounding variables cannot be entirely excluded. The study sample of community‐dwelling older adults may limit generalizability to older populations in different settings. The use of a BIA for body composition assessment may also pose limitations, such as the greater reliance on hydration status and lower precision of BIAs compared to advanced methods. A review of comorbidities related to medication use is lacking to further assess the true impact of comorbidities and medications on fall risk. The muscle strength of the hip, knee and ankle joints is also associated with fall risk, and the unavailability of data on these parameters is thought to be a limitation of fall risk estimation. Nevertheless, leg strength is not included in the sarcopenia consensus globally; based on the categorized muscle health statuses and AWGS cut‐off points of muscle health parameters for fall risk comparisons, our study did not measure this parameter.

In conclusion, our findings underscore the importance of prioritizing the management of dynapenia over sarcopenia to more effectively mitigate fall risk among community‐dwelling older adults. Moreover, older women who use sedative medications and have a history of coronary artery disease and arthritis are particularly vulnerable to increase longitudinal fall risk, warranting further assessment to tailor appropriate clinical interventions.

## Conflicts of Interest

The authors declare no conflicts of interest.

## Supporting information


**Table S1** International Classification of Diseases (ICD‐10) of considerable comorbidities or health conditions in this study.
