# Supplementary material for: Longitudinal Cohort Study Investigating Fall Risk Across Diverse Muscle Health Statuses Among Older People in the Community
Source: J Cachexia Sarcopenia Muscle. 2025 Mar 31;16(2):e13788. doi: 10.1002/jcsm.13788 (PMC11955838; doi:10.1002/jcsm.13788)
Supplement: Supplementary file 1 — Table S1 International Classification of Diseases (ICD‐10) of considerable comorbidities or health conditions in this study. [file JCSM-16-e13788-s001.docx]

Supplemental Table 1 International Classification of Diseases (ICD-10) of considerable comorbidities or health conditions in this study

| Health Conditions or Diseases | ICD-10 | Description |
| --- | --- | --- |
| Congestive Heart Failure (CHF) | I50.9 | Heart failure, unspecified |
|  | I50.1 | Left ventricular failure |
|  | I50.22 | Chronic systolic (congestive) heart failure |
|  | I50.32 | Chronic diastolic (congestive) heart failure |
| Chronic Kidney Disease (CKD) necessitating regular hemodialysis | N18.6 | End-stage renal disease (ESRD) |
|  | Z99.2 | Dependence on renal dialysis |
| Cognitive Impairment | F06.8 | Other specified mental disorders due to known physiological condition |
|  | R41.3 | Other amnesia |
|  | R41.8 | Other symptoms and signs involving cognitive functions and awareness |
|  | G31.84 | Mild cognitive impairment |
| History of chest or bone pain associated with physical exertion | R07.89 | Other chest pain |
|  | M89.9 | Disorder of bone, unspecified |
|  | R07.2 | Precordial pain |
|  | I20.9 | Angina pectoris, unspecified (if related to exertion) |
| Malignancies requiring ongoing medical treatment | C00-14 | Malignant neoplasms of lip, oral cavity, and pharynx |
|  | C15-26 | Malignant neoplasms of digestive organs |
|  | C30-39 | Malignant neoplasms of respiratory and intrathoracic organs |
|  | C40-41 | Malignant neoplasms of bone and articular cartilage |
|  | C43-44 | Melanoma and other malignant neoplasms of skin |
|  | C45-49 | Malignant neoplasms of mesothelial and soft tissue |
|  | C50 | Malignant neoplasm of breast |
|  | C51-58 | Malignant neoplasms of female genital organs |
|  | C60-63 | Malignant neoplasms of male genital organs |
|  | C64-68 | Malignant neoplasms of urinary tract |
|  | C69-72 | Malignant neoplasms of eye, brain, and other parts of the central nervous system |
|  | C73-75 | Malignant neoplasms of thyroid and other endocrine glands |
|  | C76-80 | Malignant neoplasms of ill-defined, secondary, and unspecified sites |
|  | C81-96 | Malignant neoplasms of lymphoid, hematopoietic, and related tissue |
|  | C97 | Malignant neoplasms of independent (primary) multiple sites |
| Metabolic Syndrome | E88.81 | Metabolic syndrome |
| Hypertension | I10 | Essential (primary) hypertension |
|  | I11.9 | Hypertensive heart disease without heart failure |
|  | I12.9 | Hypertensive chronic kidney disease without stage specified |
|  | I13.10 | Hypertensive heart and chronic kidney disease without heart failure and with stage 1-4 chronic kidney disease |
| Diabetes | E11 | Type 2 diabetes mellitus |
|  | E13 | Other specified diabetes mellitus |
|  | E14 | Unspecified diabetes mellitus |
|  | E11.9 | Type 2 diabetes mellitus without complications |
| Stroke | I63.9 | Cerebral infarction, unspecified |
|  | I61.9 | Nontraumatic intracerebral hemorrhage, unspecified |
|  | I64 | Stroke, not specified as hemorrhage or infarction |
|  | G45.9 | Transient cerebral ischemic attack, unspecified |
| Coronary artery disease (CAD) | I25.10 | Atherosclerotic heart disease of native coronary artery without angina pectoris |
|  | I25.11 | Atherosclerotic heart disease of native coronary artery with angina pectoris |
|  | I25.41 | Coronary artery aneurysm without rupture |
|  | I25.82 | Chronic total occlusion of coronary artery |
| Arthritis | M19 | Other and unspecified osteoarthritis |
|  | M17.9 | Osteoarthritis of knee, unspecified |
|  | M15.0 | Primary generalized (osteo)arthritis |
|  | M06.9 | Rheumatoid arthritis, unspecified |
|  | M13.9 | Arthritis, unspecified |
| Osteoporosis | M81.0 | Age-related osteoporosis without current pathological fracture |
|  | M80.0 | Age-related osteoporosis with current pathological fracture |
|  | M82 | Osteoporosis in diseases classified elsewhere |
| Depression | F32.0 | Mild depressive episode |
|  | F32.1 | Moderate depressive episode |
|  | F32.2 | Severe depressive episode without psychotic symptoms |
|  | F32.3 | Severe depressive episode with psychotic symptoms |
|  | F33.0 | Recurrent depressive disorder, current episode mild |
|  | F33.9 | Recurrent depressive disorder, unspecified |
|  | F34.1 | Dysthymia |
